# Determinants of food security status of household in west Gojjam zone, Ethiopia

**DOI:** 10.1002/fsn3.3527

**Published:** 2023-06-26

**Authors:** Chernet Worku

**Affiliations:** ^1^ Department of Agribusiness and Value Chain Management Debre Markos University Debre Markos Ethiopia

**Keywords:** food, food insecurity, food security, Heckman two‐stage model

## Abstract

Food security is based on a variety of factors, including how well agricultural production is doing, how much food we import, how many people are employed in the food industry, what public policies are put in place to improve food production strategy and food security of the household. Food is important for human beings so that everyone can have enough of it. A lack of food in the long term can lead to people becoming hungry and starving, which can be dangerous. There are many ways to boost food production and improve self‐sufficiency and food security. One way is to develop small‐scale irrigation schemes. In this study, multistage sampling was used to collect data from 400 sample rural households, and results showed that age, family size, market information, and price of the product are significant at 1% significance level, along with the non‐farm income, and irrigation participation at 5% significance level. It was also found that education is significant at 10% significance level. Governments and relevant parties need to work together to address the issues of food security by disseminating current data on the irrigation industry to boost the agricultural product, enhance irrigation facilities, and provide extension agents with training to increase the level of food security in the country.

## Graphical abstract

Food is important for human beings so that everyone can have enough of it. Food security is based on a variety of factors, including how well agricultural production is doing, how many people are employed in the food industry, what public policies are put in place to improve food production strategy and food security of the household. Therefore, this study focuses on identifying the determinants of food security of the household.

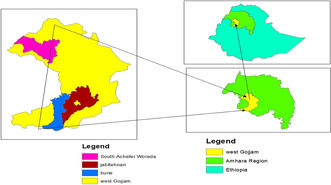

## 1 INTRODUCTION

### 1.1 Background of the study

Everyone needs food to survive and be healthy. If people do not have enough food, they can get very hungry and sick. Unfortunately, there are more and more people who do not have enough food. Last year, a lot of people in the world did not have enough food to eat. It was between 720 and 811 million people. That is, a lot of people who need help not getting enough to eat (FAO et al., 2021).

Food security is based on a variety of factors, including how well agricultural production is doing, how much food we import, how many people are employed in the food industry, what public policies are put in place, what decisions farmers make, how well our food is accessible to everyone, how much financial assistance we receive, and how wisely we use our natural resources. In order to achieve food security, we need to work together to improve all of these areas (Abide & Asfaw, 2022; Assefa & Beyene, 2023). But indirectly, food insecurity is a global problem that is threating every country on the earth. Around 720 and 811 million people in the world faced hunger in 2020 – as many as 161 million more than in 2019. Nearly, 2.37 billion people did not have access to adequate food in 2020 – an increase of 320 million people in just 1 year. This is because food insecurity means that people do not have enough food, and they cannot always get the food they want (FAO et al., 2021).

Over the past few years, Ethiopia's economic policy has been focused on agriculture in order to help reduce poverty and develop the country quickly. However, the production of food has decreased due to the high population and decreasing amount of land available (Tefera & Subaro, 2013). However, the agricultural sector can help improve the lives of rural people and ensure food security, but it is not always able to provide enough money or resources to help low‐income farmers escape poverty and food insecurity (Asmah & Avenue, 2011; World Bank, 2008).

Agriculture is a big part of the Ethiopian economy, and it contributes to 50% of the country's GDP. There are some problems with the agricultural sector, including shrinking land sizes, limited resources, and reducing soil quality. Climate change is also a big problem, affecting how much food people can produce (Welteji, 2018). In order to improve food security, we need to understand the drivers of food insecurity at the rural household level (Zerssa et al., 2021).

Most Ethiopians live in rural areas and depend on rain fed agriculture, which is very weather‐sensitive. This means that when the weather changes, many people in Ethiopia cannot afford to buy food, or they cannot get enough to eat. This is a huge problem because it keeps people from having a good life, and it is especially hard for those who live in poverty (Andersson et al., 2009). The government and other groups are working together to promote irrigation development as a way to help improve the country's overall agricultural development, and in particular, to help improve the food security of rural households. Irrigated agriculture has a positive effect on food security and income (Kebede, 2011).

The Amhara region has a lot of land that can be used to grow crops, and west Gojjam zone is especially good for irrigation because it has a lot of potential water sources. Small‐scale irrigation projects help farmers in the area to grow more crops and improve their food security, which in turn helps them improve their living conditions (WGZAO, 2021). However, most of the people in the area do not want to participate in small‐scale irrigation schemes because they rely on rain‐fed agriculture and the production is not enough to support their families. However, in the study area, the reasons for the low involvement and food insecurity problem are not entirely clear. Therefore, this study aimed to analyze food security improvement strategies and determine factors affecting food security status as well as to address the existing information gap on food security.

## 2 METHODOLOGY OF THE RESEARCH

### 2.1 Description of the study area

**FIGURE 1:**
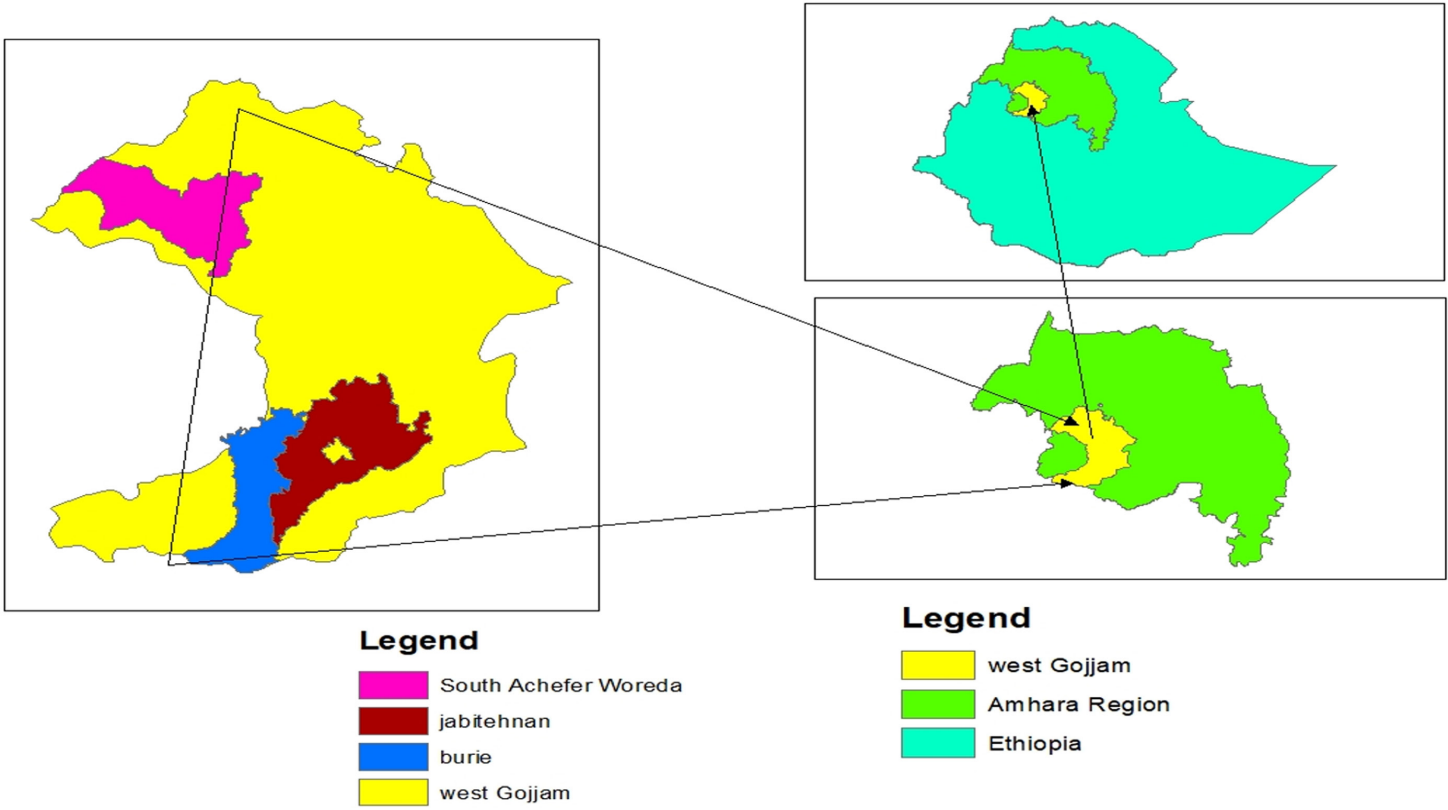
Map of the study area.

West Gojjam is a part of Ethiopia located in the southwest. It is bordered by the Abay River on the south, which separates it from the Oromia Region and the Benishabgul Gumuze Region, on the northwest by Alefa, on the east by east Gojjam, on the north by south Gondar and on the west by Awie zone. There are 14 rural woreda administrations and 2 city administrations in the study area. The map of the study area is shown in Figure 1.

### 2.2 Type, source, and method of data collection

From primary and secondary data sources, both quantitative and qualitative data were collected. For this study, to collect primary data from respondents, semi‐structured questionnaire or schedule interview was used. Additionally, focus group discussion, key informant interview, and field observation were used during primary data collection. Farmers and extension agents were interviewed utilizing a checklist in a focus group discussion. Focus group discussions were conducted in four rounds with 10 member in each district and the key informant interview was conducted in 2 rounds with 10 members. Secondary data were gathered from the office of agriculture, survey reports, annual reports, and websites. To support the interpretation of the primary data, both published and unpublished documents were examined.

### 2.3 Sample size and sampling techniques

The sample size of this study was drawn from both rural and urban living households. Three‐stage sampling procedures were employed for sampled household selection process. In the first stage, from the whole of 14 districts in west Gojjam zone, 3 districts were selected randomly. These are Burie zuria, Jabithnan, and South Achefer. In the second stage, using a simple random sampling procedure, three kebles were selected in each district by a simple random sampling technique. The administration of District and Different District Office provided lists of total households in the selected districts. Finally, using a simple random sampling procedure, the sample respondents from each district were chosen. A total sample of 400 rural and urban living households was drawn, using a probability proportional to the size of the identified households in each of the three districts (Table 1). The sample size of rural households was determined by using the following formula developed by Yamane (1967).

$$n=\frac{N}{1+N\left(e\right)2}\;n=\frac{2,106,596}{1+2,106,596\left(0.05\right)2}=400,$$

where *N* is total population = 2,106,596; *n* is sample size = 400; *e* is Error term = 0.05.

**TABLE 1:** Total population and sample size respondents.

District name	Total number of population	Total sampled households
Burie Zuria	116,076	105
Jabitehnan	148,975	134
South Achefere	179,342	161
Total	444,393	400

### 2.4 Methods of data analysis

In this study, both econometric and descriptive analysis were used to analyze the data collected from respondents.

#### 2.4.1 Descriptive statistical analysis

Descriptive statistics were used to analyze the characteristics of the sample households. Additionally, inferential statistics, such as chi‐square (*χ* ^2^‐test) and *T*‐test, were used to see if there is an association between dummy (categorical) and continuous variables with the outcomes.

#### 2.4.2 Econometric analysis

The Heckman two‐stage models, double‐hurdle model, and Tobit model are models that can help economists understand the factors that affect household income and food security status of the household (Komarek, 2010). The Tobit model, developed by economist James Tobin, is a model that describes the relationship between a non‐negative dependent variable and an independent variable (Tobin, 1958). The Tobit model assumes that people make decisions about whether or not food secured at the same time as their income. This means that factors that influence both the likelihood of food security and the level of income are taken into account (Tobin, 1958). Due to this limitation, Tobit model is not appropriate for this study.

The double‐hurdle model is a way to avoid bias when estimating the determinants of a continuous dependent variable. This model allows for all of the data to be collected in the remaining sample, no matter what the explanatory variables are. This might be appropriate in this study if there are no sample selection problems (Burke, 2009).

The Heckman two‐stage model is used to correct for bias if it arises from the sample selection. The Heckman two‐stage model helps to correct for bias if it arises from the sample selection (or selection of data) (Heckman, 1979). The first stage of the model assumes that the missing value of the dependent variable means that the dependent variable is not observed (not selected). This is used to figure out how the other variables in the model will affect the final value of the dependent variable. The second step of the model uses this information to calculate the final value of the variable (Heckman, 1979).

The Heckman model uses a probit regression to estimate the likelihood of food security status. The Heckman model uses a probit regression to estimate how likely someone is to food security (Heckman, 1979). If the inverse Mills ratio (which is computed from the probit regression) is found to be significant, the study was used to relax the assumptions of the Tobit model. Therefore, in this study, Heckman sample selection model was used.

## 3 RESULT AND DISCUSSION

### 3.1 Descriptive statistical analysis

#### 3.1.1 Demographic and socioeconomics characteristics of respondents

According to the study in Table 2, about 82% of the 400 respondents were male and 18% were female. The Chi‐square results show that sex of the household was statistically significant at the 1% significance level, which indicates that male household heads are more food secure than female household heads. Additionally, the education background of the respondents was found to be an important feature that determines the readiness of the respondents to accept new ideas and innovations. Farmers who are more educated are more likely to adopt new technology to increase their land and labor productivity. In terms of education categories, 41.5% of the respondents did not read and write, 25.5% read and write, 8.5% completed primary school, and 6.5% completed secondary school.

**TABLE 2:** Characteristics of respondents (dummy and categorical variable).

Characteristic	Item	Secured		Non‐secured		Overall	*χ* ^2^‐Value
		Freq	%	Freq	%		
Sex	Male	259	79	69	21	328	5.003**
	Female	48	33.3	24	66.7	72	
Educational status	Not read and write	166	73	54	27	193	4.899
	Read and write	101	77.7	29	22.3	130	
	Primary school	34	87.2	5	12.8	39	
	2nd school	26	83.9	5	16.1	31	
Off‐farm income	Yes	83	79.8	21	19.2	104	0.736
	No	224	75.7	72	24.3	296	
Transport facility	Yes	262	86.8	40	13.2	302	69.147***
	No	45	45.9	53	54.1	98	
Market information	Yes	287	90.3	31	9.7	318	158.468***
	No	20	24.4	62	75.6	82	
Irrigation participation	Yes	245	80.1	61	19.9	306	8.021**
	No	62	66	32	34	94	

*Note*: *** and** is statically significance at 1% significance level respectively.

Around 75% of households in Table 2 have their own transport, while 24% do not have any. The main means of transport are pack animals, which is why it is important that there are well‐functioning transport facilities available in the district. The chi‐square (*χ* ^2^) statistic shows that transportation ownership is statistically significant on food security. This helps to increase the availability of food, which in turn can improve the food security status of the households. Without access to reliable transportation, it can be difficult for farmers to sell their products or bring them to the market.

In terms of market information, about 90% of food secured household had access to market information and 10% had no access to market information from different sources. The chi‐square result shows that access to market information was statistically significant at the 1% significance level which indicates that food‐secured households get market information than non‐secured. There is no adequate and organized flow of market information in the households of the study area. Access to agricultural marketing information is an essential factor in encouraging competitive markets to easily buy and sell their food products. Reliable market information helps farmers to sell and buy their food product freely by interacting with traders and can choose a profitable mode of transaction or channels to get better benefit and become food secured.

As Table 2 indicates, out of 400 sample respondents, 306 (76.5%) participated in irrigation and the rest 94 (23.5%) did not participate in irrigation. The Chi‐square results show that irrigation participation was statistically significant at the 5% significance level which indicates that the households that participate in irrigation are more secure in food than the households that did not participate in irrigation.

As Table 3 indicates, the mean age of food secured household sample respondents was 44.8013 and food insecure household was 37.6774. The two‐tailed *T*‐test results show that the age of the household was statistically significance at the 1% significance level which indicates that the age of households increase, the household becomes food secured because the older household are more experienced that they could acquire through time.

**TABLE 3:** Mean comparison of continuous variable relation to food security.

Characteristics	Mean		Overall mean	*t*‐Value
	Food secured	Food in secured		
Age (year)	44.8013	37.6774	43.1450	−5.415
Total family size (number)	5.6894	5.3978	5.6216	−1.078***
Frequency of extension contact (number)	2.9349	1.2581	2.5450	−9.748***
Total number of livestock (number)	3.5542	3.1240	3.4541	−2.282**
Distance nearest market (min)	71.6450	93.4409	76.7125	5.193***
Farm size (ha)	1.4406	1.0788	1.3564	−4.404

*Note*: ***, **, * is statically significance at 1%, 5%, and 10% significance level respectively.

Regarding to frequency of extension contact in Table 3 the respondents who are frequently contact the extension agent to be motivate to accept new idea and innovations so that they become food secured as compared others. Farmers who are in more contact with extension agent are expected to adopt new technology to increase their productivity. The mean of extension contact that are food secured was 2.9349 and that of non‐secured was 1.2581. The two‐tailed *T*‐test results show that the frequency of extension contact of the household was statistically significant at the 1% significance level which indicates that when the farmers get different extension services by extension agent, they are more motivated to produce different types of agricultural product which help in food security.

In terms of distance to the nearest market, the respondents believed as important that they live in the nearest area and to be food secured because they sell and buy their food product easily. The mean distance of the households that are food secured was 71.6450 and that of food insecured was 93.4409. The two‐tailed *T*‐test results show that the distance to the nearest market of the household was statistically significant at the 1% significance level which indicates that when farmers live far from the market, they do not get services, which has been proved by market and may influence food security status negatively.

Table 3 indicates that the mean total livestock of food secured household was 3.5542 and that of food insecure household was 3.1240. The two‐tailed *T*‐test results show that the total livestock of the household was statistically significant at the 5% significance level which indicates that the households that have large number of livestock is more food secured as compared with households who have less number of total livestock.

### 3.2 Econometrics analysis

#### 3.2.1 Determinants of food security status

##### Age of the household

The study found that the age of the household head has a very small probability of affecting food security, but it is actually related to food security in a positive way. This means that if the age of the household head increases by 1 year, the likelihood of the household being food secure increases by 0.25%. There is a possible explanation for this, which is that an older household head is more likely to be engaged in farming activities, which can provide a steadier supply of food. Young people often prefer to live in towns and spend their time there instead of on farms, so an older head is more likely to be able to provide them with food security. The study shows that aged people's economies tend to be more stable and they tend to accumulate more wealth. Older household heads were also more likely to have a better access to land than younger heads. This is likely because older farmers usually inherit land from their grandparents, while younger farmers may have to wait for land distribution or work together with their families to get access to land. This finding agrees with the findings of Edriss and Aisay (2020) and Assefa and Beyene (2023). These factors affect food security status positively and are also in line with the study of Pakravan‐Charvadeh et al. (2021) who state that age is significantly associated with food security status. It also agrees with the findings of Bhattacharyya et al. (2023) that describe that age is significantly associated with the father's involvement in the infant and young child feeding (IYCF) practices (Table 4).

**TABLE 4:** The Hackman two‐step selection equation.

Variable	d*y*/d*x*	Std. err.	*z*	*p* > *z*
Age	0.002562***	.00069	3.72	.000
Education level	0.0105724*	.0062	1.70	.088
Family size	0.0151771***	.00341	4.45	.000
Total livestock	−0.0017803	.00544	−0.33	.744
Sex	−0.0156083	.02275	−0.69	.493
Market information	0.1221137***	.0319	3.83	.000
Price	0.0384599***	.00308	12.47	.000
None farm income	0.0505736**	.01807	2.80	.005
Extension contact	−0.0023972	.00608	−0.39	.693
Irrigation participation	0.0502467**	.01964	2.56	.011

*Note*: ***, ** and* are statistically significant at 1, 5 and 10% respectively.

##### Price of the product

If you buy a product, the price of that product affects how likely a household is to have enough food. A one birr increase in the price of a product increased the probability of food insecurity by 3.8%. This is because when the price of a product goes up, it becomes more difficult for families to afford food. However, this does not mean that food will become more expensive. When food price become high, some families may not have enough money to buy all the food they need but if the family have extra money that will be saved they may be afford more food. This is in line with the study of Worku et al. (2021), lagged price of the agricultural product has a positive impact on household income.

##### Family size of the household

The study found that a household's size (in terms of number of people) has a positive effect on their food security status at a 1% significant level. This shows that households with more people are more likely to be food secure. This is because they have more food to share, which means that there is less chance of someone in the household going hungry. However, this effect is only seen if all other things are equal. If one person in a household increases in size, the odds of the household being food secure go up by 1.52%. This is in contrast to the idea that as family size increases, food demand in the household also increases, leading to more people going hungry. The study implies that households with a high number of family members were able to share knowledge, experience, and manage agricultural activities together more easily than households with a smaller number of family members. This added value to the family lives. This finding agrees with that of Assefa and Beyene's (2023) result, which found that the family size has a positive and significant effect on food security. The finding also agrees with the finding of Pakravan‐Charvadeh, Flora, et al. (2022) and Pakravan‐Charvadeh, Vatanparast, et al. (2022). Household size has a direct and positive asocial implication, which means that in rural areas when more people are living in a house, they tend to use their food more efficiently and get more nutrients from it.

##### Access to market information

The probability of a household's food security status is affected by how easily they can get information about the market. The market information of the household is positively significant at the 1% level of probability. This means that if one unit of access to market information is added, the probability of food security goes up by 12.2%. However, this effect is only seen when all other factors are held constant.

##### Non‐farm income

The coefficient of non‐farm income had a positive impact on the probability of food security status of households and it is significant at a 1% significant level. This means that households with more non‐farm income are more likely to have food security than those who do not have other income. This is because these households can often afford to buy more food and have extra cash to spend on other inputs, which can help them produce more food. The farm households in this study have a higher chance of being food secure if they have more money. This is because when their non‐farm income increases, it also means they can afford to buy more food, which in turn makes them more secure. The farm households in this study are more likely to have extra money to invest in other things that can help them stay safe from hunger. When their income goes up by 1 unit, the chances of them being food secure go up by 5.1%. This finding agrees with that of Fikire and Zegeye (2022) and Assefa and Beyene (2023). Non‐farm income has a positive effect on the food security of the household. Additionally, it is in line with the finding of Pakravan‐Charvadeh, Flora, et al. (2022) and Pakravan‐Charvadeh, Vatanparast, et al. (2022). The study showed that income has a significant association with food insecurity status.

##### Education level of the household

The educational attainment of the head of the household is one of many factors that can affect a household's food security. It was found to be significant at a 10% level of significance, so education could help a lot to improve the food security status of households. It is usually expected that people with more education will be better able to earn a good income and manage their food resources more efficiently. This is because educated people are more likely to be interested in getting information about farming and other ways to make money, and they are also more likely to be interested in taking agricultural or livestock extension services, and trying out techniques to protect their land and water supplies. This means that when people learn more, they are more likely to have enough food to eat and become food secured. This finding agrees with that of Edriss and Aisay (2020) and Assefa and Beyene (2023), and the results found that education has a positive and significant effect on food security and are also in line with the study of Khakpour et al. (2021) who stated that the level of education has a significant and positive association with food security status of the household, and also agrees with the study of Gaiser et al. (2023) who state that lower level of education has a significant association in malnutrition which causes underweight.

##### Participation in irrigation

The variable had a positive relationship with food security status and was significant at a 5% probability level. This means that households who had more hectares of irrigation land were more likely to achieve their food security status and cope with food insecurity factors. Irrigation helps farmers to produce crops even when there is no enough rain, and this can help improve food security. That is because irrigation uses a lot of water to help plants grow. It has a big impact on people's food security status because it helps us get food even when the weather is not cooperating. This finding is in line with that of Getaneh et al. (2022). The results show that irrigation has a positive and significant effect on food security.

## 4 CONCLUSION AND RECOMMENDATION

There are a lot of different things that can affect a household food security status, and some of those things are pretty important. So, to increase household food security status and their income, we have to pay close attention to things like household education status, off‐farm income, and access to market information.

The study found that by targeting the rural household enhancement of their education level, improving extension services, establishing irrigation facilities to help them participate in irrigation, and using better production methods, they can increase their income and become secured in food.

The Heckman two‐step selection equation model found that different factors have influenced household about whether to be secured in food or not. This included providing education and services to the producers to help them make better decisions about irrigation technology and improve their livening standard.

The study found that farmers who did not get market information are less likely to be secured in food, because they cannot sell and buy their food products if they cannot get it to the market. However, the level of education of household affects food security status, and increasing awareness of this among households is one way to encourage more households to become food secured. Additionally, off‐farm income, the size of the farm, and the age of the household also affect food security status. Stakeholders need to focus on improving access to transportation, markets, and other infrastructure so that more farmers can sell and buy their crops that help in food security.

## FUNDING INFORMATION

The author did not receive any funding for this study and it was entirely supported by the author himself.

## CONFLICT OF INTEREST STATEMENT

The researcher declares no conflict of interests.

## ETHICS STATEMENT

Ethical clearance was obtained from the Research Review Committee of Debre Markos University Burie campus and this study was approved by the Research Committee of Debre Markos University Burie campus.

## Data Availability

The author wants to declare that he can submit the data at any time based on publisher's request. The datasets used and/or analyzed during the current study will be available from the author on reasonable request.
